# Toll-like receptor and C-type lectin receptor agonists attenuate osteogenic differentiation in human dental pulp stem cells

**DOI:** 10.1186/s12903-024-03894-7

**Published:** 2024-01-31

**Authors:** Wajathip Bulanawichit, Chanakarn Sinsareekul, Chatvadee Kornsuthisopon, Ajjima Chansaenroj, Vorapat Trachoo, Nunthawan Nowwarote, Thanaphum Osathanon

**Affiliations:** 1 Center of Excellence for Dental Stem Cell Biology, Department of Anatomy, Faculty of Dentistry, Chulalongkorn University, Bangkok, Thailand; 2 Department of Operative Dentistry, Faculty of Dentistry, Chulalongkorn University, Bangkok, Thailand; 3 Department of Oral and Maxillofacial Surgery, Faculty of Dentistry, Chulalongkorn University, Bangkok, Thailand; 4 Centre de Recherche des Cordeliers, Université Paris Cité, Sorbonne Université, INSERM UMR1138, Molecular Oral Pathophysiology and Department of Oral Biology, Faculty of Dentistry, Université Paris Cité, Paris, France

**Keywords:** Dental pulp cells, Osteogenic differentiation, Cell viability, Toll-like receptor

## Abstract

**Background:**

This study aimed to investigate the effects of various toll-like receptor (TLR) and C-type lectin receptor (CLR) ligands on osteogenic differentiation in human dental pulp stem cells (hDPSCs).

**Methods:**

hDPSCs were cultured and treated with various concentrations (0.01, 0.1, 1.0, and 10 µg/mL) of TLR or CLR agonists (*PG*-LPS, *E.coli* LPS, poly(I:C), Pam3CSK4, Furfurman, and Zymosan). Cell viability was determined by MTT assay. The effects of TLR and CLR agonists on osteogenic differentiation of hDPSCs were measured by alkaline phosphatase (ALP) activity, Alizarin Red S staining, and Von Kossa staining. In addition, the mRNA expression of osteogenesis-related genes (*ALP*, *COL1A1*, *RUNX2, OSX, OCN* and *DMP1*) was examined by RT-qPCR. A non-parametric analysis was employed for the statistical analyses. The statistically significant difference was considered when *p* < 0.05.

**Results:**

Treatment with TLR and CLR agonists was associated with an increase in hDPSCs’ colony-forming unit ability. Compared with the control group, TLR and CLR agonists significantly inhibited the osteogenic differentiation of hDPSCs by decreasing the ALP activity, mineralised nodule formation, and mRNA expression levels of osteogenesis-related genes (*ALP, COL1A1, RUNX2, OSX*, *OCN* and *DMP1*). The inhibition of TRIF but not Akt signalling rescued the effects of TLR and CLR agonist attenuating hDPSCs’ mineralisation.

**Conclusions:**

The activation of TLRs or CLRs exhibited an inhibitory effect on osteogenic differentiation of hDPSCs via the TRIF-dependent signalling pathway.

**Supplementary Information:**

The online version contains supplementary material available at 10.1186/s12903-024-03894-7.

## Introduction

Pulpitis is an inflammatory condition affecting the dental pulp, primarily caused by bacterial infection. Pattern recognition receptors (PRRs) recognise the invading pathogen via recognised pathogen-associated molecular patterns (PAMPs). Common PRRs comprised of toll-like receptors (TLRs), nucleotide-binding oligomerization domain-like receptors (NLRs), RIG-I-like receptors (RLRs), C-type lectin receptors (CLRs), and Absent in melanoma-2-like receptor (ALRs) [1]. Currently, there are 10 functional human TLRs identified. These receptors are categorised based on their cellular localisation and their specific recognition of PAMPs [2]. Upon the recognition of PAMPs, TLR initiates intricate signalling cascades that stimulate the expression of proinflammatory cytokines and activate immune responses.

Dental pulp stem cells (DPSCs) are specific mesenchymal stem cells (MSCs) that exist in human dental pulp tissues [3]. They exhibit remarkable regenerative potential attributed to their capacity for self-renewal and multi-lineage differentiation [4]. In response to pulpal injury or infection, DPSCs encounter microbes and their components, for example, gram-negative bacteria and fungi. TLRs recognise PAMPs of bacteria, viruses, and fungi, while CLRs mainly recognise PAMPs of fungi [1]. DPSCs expressed a variety of functional TLRs with upregulation of TLR2, TLR3, and TLR4 [5], which play a key role in the advancement of pulpal disease. It has become apparent that pulpal stimulation by TLR-specific agonists may significantly influence the migratory, proliferative, and differentiation potentials of the DPSCs [6].

Lipopolysaccharide (LPS), polyinosinic:polycytidylic acid (poly(I:C)), palmitoyl-3-cysteine-serine-lysine-4 (Pam3CSK4), Furfurman, and Zymosan have been reported as the specific ligand to activate TLRs-induced cellular signalling in hDPSCs. LPS mainly interacts with TLR4 [7]. Poly(I:C) binds with TLR3 to exert its mechanism of action [8]. Pam3CSK4 is recognised as a TLR1/2 agonist [9]. Zymosan collaborates with TLR2 to mediate the innate immune response [10]. Furfurman binds to Dectin-2 and Dectin-1. Several studies investigate the role of TLR agonists in the proliferation and osteoblastic differentiation of hDPSCs. For example, it has been reported that cell proliferation was decreased while osteogenic differentiation remained unaffected following treatment with LPS [11]. However, a contradictory result was observed in other studies [12, 13]. In addition, hDPSCs exhibit distinct inflammatory mediators in response to stimulation with bacterial LPS, Pam3CSK4, and poly(I:C), which affect the differentiation of hDPSC and coordinate tissue repair [14–17].

From the result of previous studies, it can be implied that different microbial compositions (bacteria and fungi) of the caries lesion may exert distinct influences on the inflammatory response of the pulp [18, 19]. However, there are some contradictory data regarding the roles of different TLRs in the differentiation of hDPSCs. In addition, the effects of Zymosan and Furfurman on the interactions of hDPSCs have only been investigated in a limited number of studies. Therefore, this study aimed to investigate the effect of various concentrations of TLR and CLR agonists (*PG*-LPS, *E.coli* LPS, Poly (I:C), Pam3CSK4, Furfurman, and Zymosan) on the colony-forming unit ability and osteogenic differentiation of hDPSCs. The null hypothesis is that TLR and CLR agonists do not affect osteogenic differentiation in hDPSCs.

## Materials and methods

### hDPSCs isolation

The cell isolation protocol was approved by the Human Research Ethics Committee, Faculty of Dentistry, Chulalongkorn University (Approval number 037/2021). The healthy teeth from the patients aged 18–30 years old, extracted according to the patient’s treatment plan (for example, embedded and impacted teeth), were obtained for cell isolation. The teeth with pathological conditions were excluded from cell collection. Cells were isolated using the explantation technique. Briefly, tissues were cut into small pieces and placed on 35-mm tissue culture plates, allowing cell outgrowth for 7 days. The isolated cells were cultured in a growth medium composed of Dulbecco’s modified Eagle Medium (DMEM) (Gibco, USA) supplemented with 10% fetal bovine serum, 1% L-glutamine (Gibco, USA), 1% Antibiotic-Antimycotic (Gibco, USA) and incubated at 37 °C in 5% CO_2_. After reaching confluence, the cells were trypsinised and subcultured at a 1:3 ratio using trypsin/EDTA solution (Gibco, USA). The culture medium was changed every 48 h. Cells from passages 3–6 were employed in the present study.

### Mesenchymal stem cell characterisation

Cells at passages 2–3 were harvested. The single-cell suspension was prepared for fluorescence-conjugated antibody staining. Cell suspension was incubated with FITC conjugated anti-human CD44 (BD Bioscience, USA), PE-conjugated anti-human CD105 (Immuno Tools, Germany), FITC conjugated anti-CD90 (Abcam, USA), or PerCP-conjugated anti-CD45 (Abcam, USA) for 30 min. The surface marker staining was evaluated using a FACSCalibur flow cytometer with CellQuest software (BD Bioscience, USA). The mean fluorescence was evaluated.

For multipotential differentiation ability, cells were seeded in 24 well plates at the density of 25,000 cells per well in a normal growth medium (GM). After 24 h, the medium was changed to an osteogenic induction medium (OM) or an adipogenic induction medium (AM). The medium was replaced with the fresh medium every 48 h. The mineral deposition and intracellular lipid droplet accumulation were evaluated using Alizarin Red S staining on day 14 and Oil-Red O staining on day 16, respectively. The adipogenic medium contained 0.1 mg/ml insulin (Sigma-Aldrich, USA), 1 µM dexamethasone (Sigma-Aldrich, USA), 1 mM IBMX (Thermo Fisher Scientific, USA), and 0.2 mM indomethacin (Sigma-Aldrich, USA). An osteogenic differentiation medium comprised of growth medium supplemented with 5 mM β-glycerophosphate (Sigma-Aldrich, USA), 50 µg/ml L-ascorbic acid (Sigma-Aldrich, USA), and 250 nM dexamethasone (Sigma-Aldrich, USA).

### Treatment protocol

The cells were treated with various concentrations (0.01-10 µg/ml) of TLR agonists: *PG*-LPS (Invivogen, CA, USA), *E.coli* LPS (Invivogen, CA, USA), Poly (I:C) (Invivogen, CA, USA), Pam3CSK4 (Invivogen, CA, USA), and Zymosan (Invivogen, CA, USA) or CLR agonist: Furfurman (Invivogen, CA, USA). In some experiments, cells were pretreated with 10 µM TRIF inhibitor or 10 µM AKT inhibitor (Calbiochem, USA) for 30 min prior to TLR agonists exposure.

### Cell viability test

Cells (*n* = 4) were seeded in 24-well plates at a density of 25,000 cells per well and maintained in a normal growth medium for 1, 3, and 7 days. At the designated time points, the culture medium was removed, and the cells were incubated with 0.5 mg/ml MTT solution (3-(4,5-dimethylthiazol-2-yl)-2,5-diphenyl-tetrazolium bromide) for 30 min at 37 °C in 5% CO_2_. Cells were then washed with phosphate-buffered saline to remove the MTT solution. The precipitated formazan crystals were solubilised in dimethyl sulfoxide (DMSO; Carlo Erba, 900 µl per well) and glycine buffer (pH = 10, 125 µl per well). The solubilised solution was collected, and the absorbance was measured at 570 nm using a microplate reader (Biotek ELX800, USA). The absorbance values were then normalised to the control condition at day 1.

### Colony forming unit assay

Cells (*n* = 4) were seeded at a density of 500 cells in a 35 mm tissue culture plate and maintained in a growth medium for 14 days. The culture medium was changed every 2 days. At day 14, cells were fixed with 4% paraformaldehyde and washed with deionised water (DI). Colonies were stained with Coomassie blue (Sigma-Aldrich, USA).

### Alkaline phosphatase (ALP) enzyme activity

Cells (*n* = 5) were seeded in 24-well plates at a density of 25,000 cells per well and maintained in a normal growth medium for 24 h. Subsequently, the medium was changed to an osteogenic induction medium. The culture medium was changed every 48 h. On day 7, cells were then fixed with 4% paraformaldehyde for 10 min and washed with DI, then incubated with NBT/BCIP solution (Roche, Germany) per well for 15 min at room temperature, according to the manufacturer’s protocol. After staining, cells were washed with DI and air-dried. The ALP-stained cells were observed using an inverted microscope (Olympus, USA).

### Mineralisation assay

Cells (*n* = 4) were seeded in 24-well plates at a density of 25,000 cells per well and maintained in a normal growth medium for 24 h. Subsequently, the medium was changed to an osteogenic induction medium. The culture medium was changed every 48 h. Cells were then fixed with 4% paraformaldehyde for 10 min and washed with DI. Cells were stained at room temperature with a 0.5% (w/v) Alizarin Red S solution with gentle agitation. The excess staining was washed with DI. The staining was observed using an inverted microscope (Olympus, USA). The stain was solubilised in 10% cetylpyridinium chloride monohydrate in 10 mM sodium phosphate (Sigma-Aldrich, USA). The absorbance was measured at 570 nm using a microplate reader (Biotek ELX800, USA). The absorbance values were then normalised to the control condition. As for Von Kossa staining, cells (*n* = 4) were fixed with 4% paraformaldehyde for 10 min and washed with DI. At room temperature, the cells were stained with a 5% silver nitrate solution under ultraviolet light for 5 min. The stained cells were observed using an inverted microscope (Olympus, USA).

### Quantitative polymerase chain reaction (qPCR)

Cells (*n* = 4) were seeded in 24-well plates at a density of 25,000 cells per well and maintained in a normal growth medium for 24 h. Subsequently, the medium was changed to an osteogenic induction medium. The culture medium was changed every 48 h. Total RNA was extracted with RiboEx™ reagents (GeneAll, Korea) on day 7. The quality and quantity of isolated RNA was evaluated using Nanodrop. The cDNA was synthesised with iScript RT Supermix (Bio-Rad, USA) from 1 µg of the isolated RNA. For quantification of mRNA expression, qPCR amplification was performed using CFX Connect™ Real-Time PCR (Bio-Rad, USA), and PCR products were detected using FastStart Essential DNA Green Master (Bio-Rad, USA). The amplification profile was 95 °C/20 s, 60 °C/20 s, and 72 °C/20 s for 45 cycles. A melting curve analysis was performed for the evaluation of PCR product specificity. Target gene expression was normalised by the levels of *GAPDH*. The forward and reverse primers are in Table S1.

### Statistical analysis

Data were analysed, and graphs were generated using GraphPad Prism 8 (GraphPad Software, CA, USA). Data were expressed as the mean ± standard error of the mean (SEM). In each experiment, cells from at least four different donors were used without pooling. The statistical comparison was performed using the Kruskal-Wallis test, followed by a pairwise comparison test for three or more group comparisons. A pairwise comparison was performed between the treatment groups and the control group. For a two-group comparison, the Mann-Whitney U test was used. A value of *p* < 0.05 was considered statistically significant. Each dot on the graph represented each normalisation of the raw data value.

## Results

### Stem cell characterisation

**Fig. 1:**
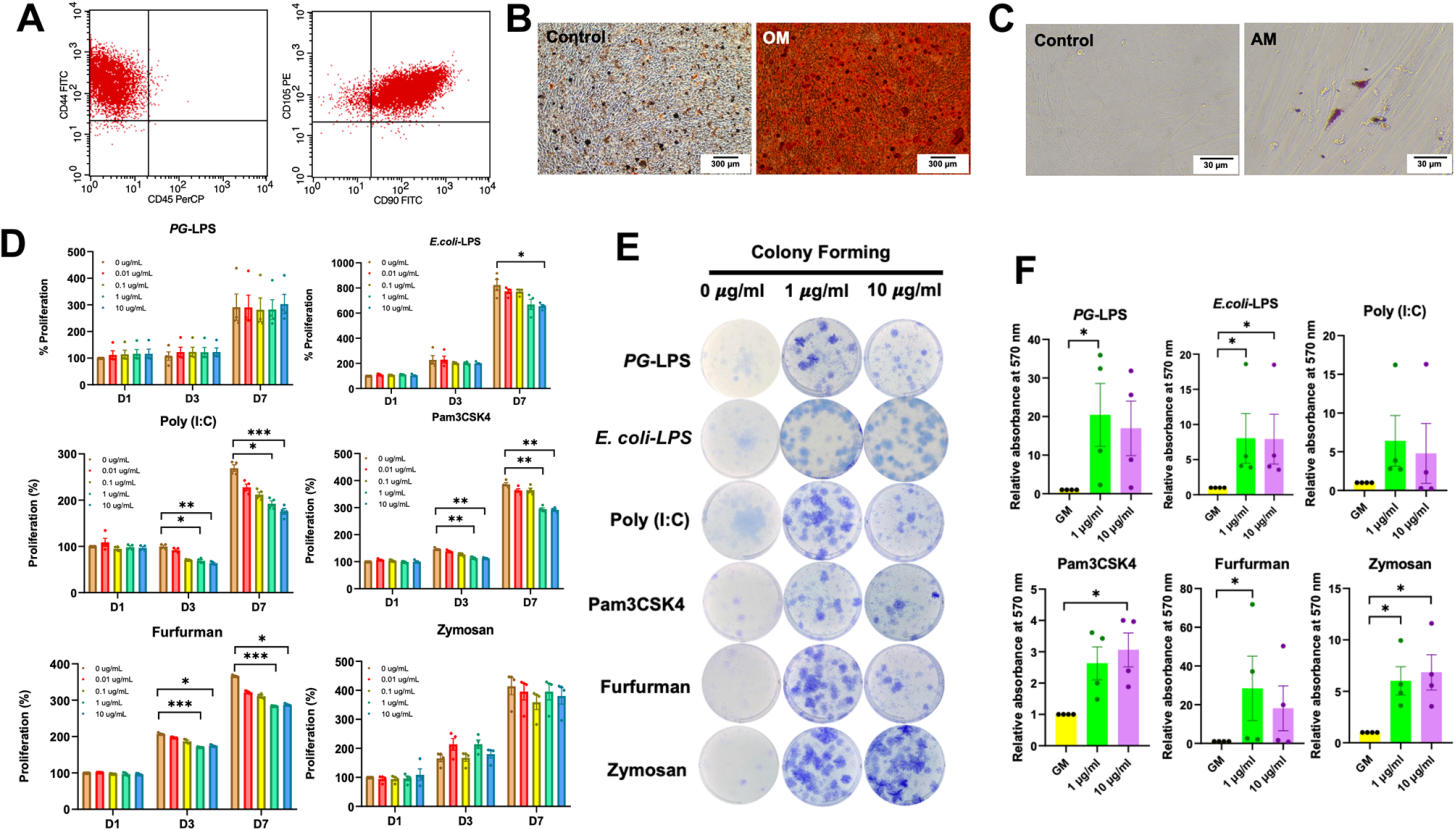
Characterisation of cells harvested from dental pulp tissues. Stem cell surface markers were examined using flow cytometry (**A**). The differentiation ability towards osteogenic (**B**) (scale bar, 300 μm) and adipogenic lineages (**C**) (scale bar, 30 μm) was examined using Alizarin Red S staining and Oil Red O staining, respectively. Effect of various TLR agonists on cell proliferation of hDPSCs. Cells were treated with *PG*-LPS, *E.coli* LPS, Poly (I:**C**), Pam3CSK4, Furfurman, and Zymosan (0, 0.01, 0.1, 1, and 10 µg/ml) for 1, 3, and 7 days. Cell proliferation was observed using an MTT assay (**D**). Cells were treated with TLR agonists at 1 and 10 µg/ml and cultured for 14 days. Colony formation was examined using Coomassie blue staining (**E**). The relative absorbance of the solubilised dye was evaluated (**F**). Bars indicate a statistically significant difference between groups (*p* < 0.05). GM; normal growth medium, OM; osteogenic induction medium, AM; adipogenic induction medium

The cells harvested from dental pulp tissue exhibited a spindle-shaped and fibroblast-like morphology. Surface protein analysis confirmed the presence of mesenchymal stem cell-related surface markers (CD44, CD90, and CD105). In contrast, the hematopoietic cell marker, CD45, was notably absent (Fig. 1A). Differentiation ability towards osteogenic and adipogenic lineages was performed. In the osteogenic induction medium, mineral deposition manifested as distinct red nodules stained by Alizarin Red S staining (Fig. 1B). The cells maintained in the adipogenic induction medium demonstrated an intracellular lipid accumulation, visualised through Oil Red O staining (Fig. 1C).

### TLR and CLR agonists reduced hDPSCs cell viability but enhanced colony-forming unit ability

MTT assays were performed with 0.01, 0.1, 1, and 10 µg/mL of *PG*-LPS, *E.coli* LPS, Poly (I:C), Pam3CSK4, Furfurman, and Zymosan at day 1, 3 and 7. The results showed that cell viability was significantly decreased when cells were treated with high concentration of *E.coli* LPS, Poly (I:C), Pam3CSK4, and Furfurman at day 7 (*p* < 0.05) (Fig. 1D). Poly (I:C), Pam3CSK4, and Furfurman (1 and 10 µg/ml) significantly reduced cell viability of hDPSCs at day 3 (*p* < 0.05). However, there was no statistically significant difference in cell viability when cells were treated with *PG*-LPS and Zymosan.

On the contrary, TLR and CLR agonists markedly promoted the colony-forming unit ability of hDPSCs. TLR and CLR agonist-treated hDPSCs (1 µg/ml) displayed a significant increase in colony-forming efficiency compared to the control group, except for Poly (I:C) and Pam3CSK4 (*p* < 0.05). Moreover, *E.coli* LPS, Pam3CSK4, and Zymosan-treated cells at 10 µg/ml exhibited a statistically significant higher colony-forming efficiency in comparison with the control group (*p* < 0.05). No significant difference was observed between the 1 µg/ml and 10 µg/ml concentrations (Fig. 1E and F).

### TLR and CLR agonists impaired the osteogenic differentiation of hDPSCs

**Fig. 2:**
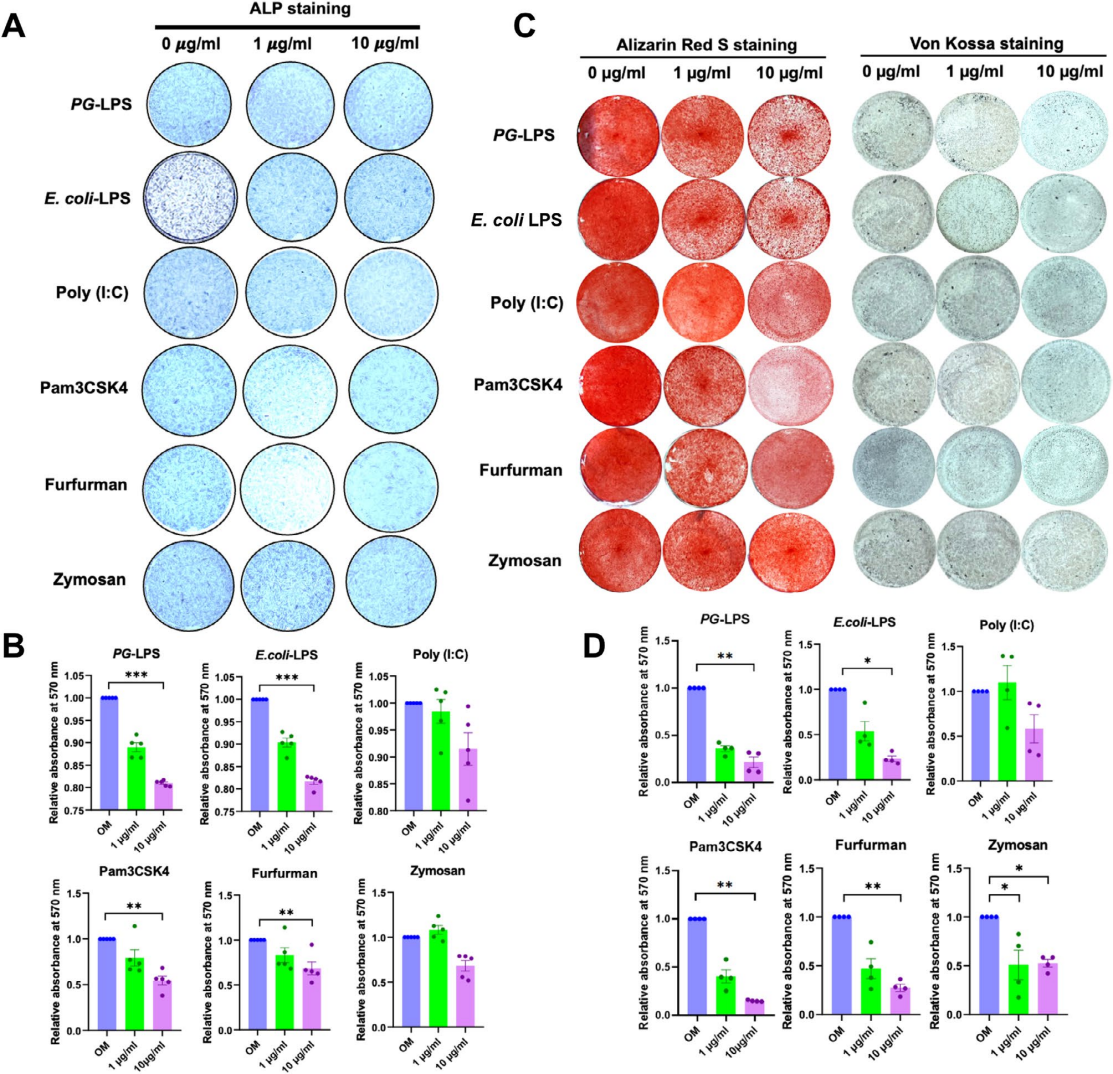
Effect of TLR and CLR agonists on the osteogenic differentiation of hDPSCs. Cells were treated with TLR or CLR agonists at 1 and 10 µg/ml in an osteogenic induction medium (OM). Alkaline Phosphatase Activity (ALP) activity was examined on day 7 (**A**). The relative absorbance of the solubilised ALP staining was measured (**B**). The mineralisation was shown by Alizarin Red S staining and Von Kossa staining on day 14 (**C**). The relative absorbance of the solubilised alizarin red dye was measured at 570 nm (**D**). Bars indicate a statistically significant difference between groups (*p* < 0.05)

**Fig. 3:**
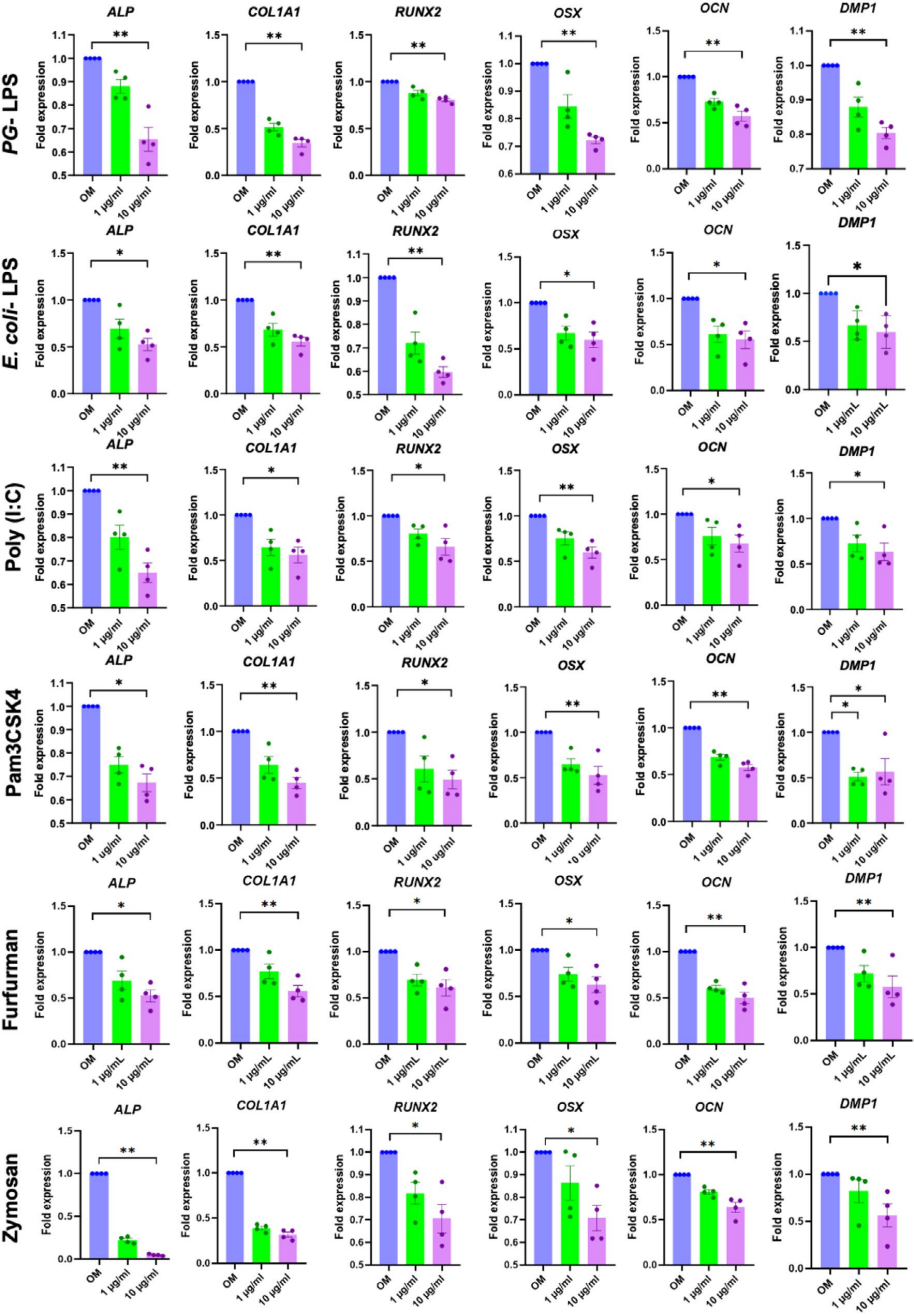
Effect of TLR agonists on the osteogenic differentiation of hDPSCs. Cells were treated with TLR agonists at 1 and 10 µg/ml in an osteogenic induction medium (OM). The mRNA expression of osteogenic-related genes was examined using qPCR. Bars indicate the statistically significant difference between groups (*p* < 0.05)

To investigate the effect of TLR and CLR agonists on osteogenic differentiation, hDPSCs were treated with *PG*-LPS, *E.coli* LPS, Poly (I:C), Pam3CSK4, Furfurman, or Zymosan at concentrations of 1 and 10 µg/ml in osteogenic medium. ALP enzymatic activity was downregulated in a dose-dependent manner at day 7 (Fig. 2A). A significant decrease in ALP enzymatic activity was observed in the *PG*-LPS*-, E.coli* LPS-, Pam3CSK4-, and Furfurman-treated groups at a concentration of 10 µg/ml compared with the control (*p* < 0.05) (Fig. 2B). Further, mineralisation was dramatically inhibited in a dose-dependent manner at day 14 (Fig. 2C and D). A significant downregulation of mineral deposition was noted in the *PG*-LPS*-, E.coli* LPS-, Pam3CSK4-, Furfurman, and Zymosan-treated groups (*p* < 0.05). Moreover, 10 µg/ml of all treatments significantly decreased the mRNA expression of key osteogenesis-related regulators and effector genes (*ALP*, *COL1A1, RUNX2, OSX*, and *DMP*) as compared with the control (*p* < 0.05) (Fig. 3).

### TRIF inhibition rescued TLR and CLR agonist-attenuated mineralisation in hDPSCs

**Fig. 4:**
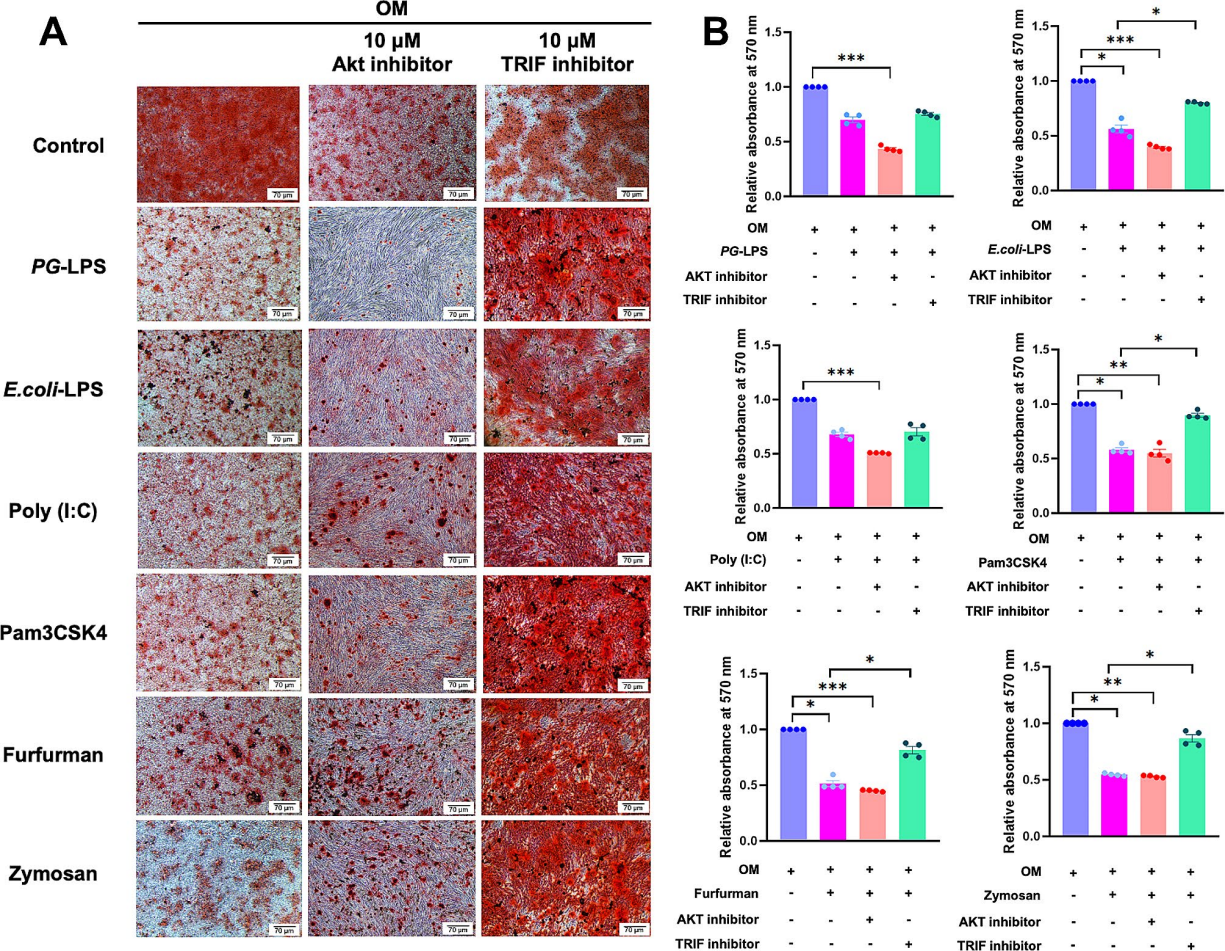
To investigate the potential pathways involved in TLRs-attenuated osteogenic differentiation of hDPSCs, cells were pretreated with an Akt inhibitor or TRIF inhibitor 30 min prior to TLR agonist exposure and maintained in osteogenic induction medium (OM). Mineralisation was examined by Alizarin Red S staining (**A**). The relative absorbance of the solubilised alizarin red dye was measured at 570 nm (**B**). Bars indicate the statistically significant difference between groups (*p* < 0.05)

To investigate the potential pathways involved in TLRs/CLRs-attenuated osteogenic differentiation of hDPSCs, cells were pretreated with an Akt inhibitor or TRIF inhibitor 30 min prior to TLR/CLR agonist exposure (10 µg/ml). The result showed that pretreatment with a TRIF inhibitor prior to TLR/CLR agonist exposure abolished the TLR/CLR agonist-attenuated mineralisation (Fig. 4A and B). Pretreatment with an Akt inhibitor failed to rescue the effects of TLR/CLR agonist-attenuated mineralisation.

## Discussion

The growing interest in utilising hDPSCs for cellular therapy highlights the need to consider the potential impact of microbial components on hDPSCs growth and differentiation. Exposure to environments enriched with bacterial endotoxins may have various effects on hDPSCs properties, including self-renewal, differentiation potential, cytokine production, and extracellular matrix composition [20]. Our results demonstrated that exposure of hDPSCs to TLR/CLR agonists negatively influences osteogenic differentiation.

LPS from gram-negative bacteria mainly interacts with TLR4 [7]. Poly(I:C), a synthetic analogue of dsRNA, binds with TLR3 to exert its mechanism of action [8]. Pam3CSK4, a synthetic triacylated lipopeptide, is recognised as a TLR1/2 ligand [9]. Furfurman binds to CLRs, namely Dectin-2 and Dectin-1 (high concentration). Zymosan collaborates with TLR2 to mediate the innate immune response [10] and it is also able to bind to Dectin-1. With the complex nature of binding efficacy, the interpretation of the results should be done with caution because the phenomenon could occur from the activation of multiple types of receptors.

Cell proliferation plays an important role in tissue repair, which is significantly influenced by the engagement of specific PRRs agonists. Previous research reported the contradictory effect of TLR agonists on cell proliferation, encompassing instances of both stimulation [21, 22] and reduction in proliferative capacity [23], while other studies have not observed any significant effect [24–26]. In the present study, *E.coli* LPS, Poly (I:C), Pam3CSK4, and Furfurman in tested concentrations did not affect the cell viability at day 1; however, hDPSCs cell viability was attenuated as compared with the control at later time points.

The present study showed that TLR and CLR agonist-treated cells enhanced the colony formation ability of hDPSCs. Our results were inconsistent with previous studies, which showed that dental pulp stem cells (hDPSCs) derived from inflamed tooth pulp exhibit a diminished ability to form cell colonies [27, 28]. Moreover, the results of the clonogenic assay give the opposite results of the MTT assay. The possible explanation includes variations in the testing methodology. The clonogenic assay evaluates cell survival by measuring the ability of a single cell to form a colony. In contrast, the MTT assay measures mitochondrial activity, referring to cell metabolic activity. This method is generally used to determine the increase in cell number. However, it is only considered to be an indirect approach. The reduction of cellular metabolic activities can result from several factors, including cell death. The colony-forming unit assay provides information regarding the cells’ ability to survive and grow but at a single-cell level. Further, the cell cycle should be used to evaluate the role of these TLR agonists on cell proliferation.

Recent studies have yielded contradictory findings regarding the impact of TLR and CLR agonists on cell differentiation. Previous studies reported that *E.coli* LPS-mediated TLR4 activation promoted osteogenic differentiation as indicated by an increase in mineralised nodule formation and odontoblastic-related mRNA expression [13, 24]. On the contrary, other studies demonstrated that *PG*-LPS and *E.coli* LPS exhibited inhibitory effects on osteoblastic differentiation of human periodontal ligament stem cells and murine dental pulp-derived cell lines [21, 26, 29, 30]. Further, exposure of dental pulp cells to the cell wall mannan of Candida species, a CLR ligand, led to the reduction of mineralisation [18]. In accordance with these latter studies, our result showed that different concentrations of TLR and CLR agonists used in the present study (*PG*-LPS, *E.coli* LPS, poly(I:C), Pam3CSK4, Furfurman, and Zymosan) impaired the osteogenic differentiation of hDPSCs, as evidenced by the reduction in both ALP activity and mineralised nodule formation. Moreover, the mRNA expression levels of osteogenesis marker genes (*ALP, COL1A1, RUNX2, OSX, OCN* and *DMP*) were downregulated. This data discrepancy could be attributed to the concentration of TLR and CLR agonists used in the present study.

The stimulation of TLR agonists triggers the activation of two major downstream signalling pathways: the myeloid differentiation factor 88- (MyD88-) dependent and TIR (Toll-interleukin receptor) domain-containing adapter-inducing interferon- (IFN-) β- (TRIF-) dependent pathways [31]. In addition to the TRIF-dependent pathway, other downstream signalling pathways, such as PI3K-Akt, have also been reported to play an important role in the osteogenic differentiation of human dental pulp cells and murine tendon stem cells [13, 32]. Thus, in the present study, we employed the pharmacologic inhibitor of TRIF or Akt to investigate the effect of TRIF/Akt signalling on the osteogenic differentiation of hDPSCs after TLR or CLR agonist stimulation. Our result demonstrated that TRIF inhibition rescued the inhibitory effects of TLR and CLR agonists on the osteogenic differentiation by hDPSCs. These findings imply that TRIF plays a crucial role during the differentiation phases of hDPSCs within the infected dental pulp.

The limitation of this study is primary cell multiplicity, which may react differently despite similar chemical substances being exposed. Furthermore, in vitro cellular responses may differ when cultured *in vivo.* Hence, it is essential to investigate animal models to obtain more efficient data on TLR and CLR agonists attenuated osteogenic differentiation.

## Conclusion

The present study demonstrated that TLR and CLR activation promoted colony formation. Further, the osteogenic differentiation was attenuated in a dose-dependent manner. This inhibitory effect of TLR activation on osteogenic differentiation occurs through TRIF but not Akt signalling. This knowledge elucidates the role of various TLR and CLR agonists on hDPSCs, which potentially advances our comprehension of the management strategies for the diseased pulp conditions.

### Electronic supplementary material

Below is the link to the electronic supplementary material.

Supplementary Material 1

## Data Availability

The datasets used and/or analysed during the current study are available from the corresponding author upon reasonable request.
