# Supplementary material for: Toll-like receptor and C-type lectin receptor agonists attenuate osteogenic differentiation in human dental pulp stem cells
Source: BMC Oral Health. 2024 Jan 31;24:148. doi: 10.1186/s12903-024-03894-7 (PMC10832253; doi:10.1186/s12903-024-03894-7)
Supplement: Supplementary file 1 — Supplementary Material 1 [file 12903_2024_3894_MOESM1_ESM.docx]

**Supplementary Table**

Table S1 The oligonucleotide sequences

| **Gene** | **Sequences** |
| --- | --- |
| ***RUNX2*** | 5’-ATG ATG ACA CTG CCA CCT CTG A -3’  5’-GGC TGG ATA GTG CAT TCG TG-3’ |
| ***OSX*** | 5’-GCC AGA AGC TGT GAA ACC TC-3’  5’-GCT GCA AGC TCT CCA TAA CC-3’ |
| ***ALP*** | 5’-CGA GAT ACA AGC ACT CCC ACT TC-3’  5’-CTG TTC AGC TCG TAC TGC ATC ATG TC-3’ |
| ***COL1A1*** | 5’-GTG CTA AAG GTG CCA ATG GT-3’  5’-ACC AGG TTC ACC GCT GTT AC- 3’ |
| ***DMP1*** | 5’-CTC CTT TAT GTG ACA ACT GC-3’  5’-ATG CCT ATC ACA ACA AAC C-3’ |
| ***OCN*** | 5’-CTT TGT GTC CAA GTA GGA GG-3’  5’-CTG AAA GCC GAT GTG GTC AG-3’ |
| ***GAPDH*** | 5’-CAC TGC CAA CGT GTC AGT GGT G -3’  5’- GTA GCC CAG GAT GCC GAG-3’ |
